# Regression of fibrosis by cilostazol in a rat model of thioacetamide-induced liver fibrosis: Up regulation of hepatic cAMP, and modulation of inflammatory, oxidative stress and apoptotic biomarkers

**DOI:** 10.1371/journal.pone.0216301

**Published:** 2019-05-08

**Authors:** Sally A. El Awdan, Rehab F. Abdel Rahman, Heba M. Ibrahim, Rehab R. Hegazy, Salma A. El Marasy, Manal Badawi, Mahmoud S. Arbid

**Affiliations:** 1 Pharmacology Department, Medical Division, National Research Centre, Giza, Egypt; 2 Pathology Department, Medical Division, National Research Centre, Giza, Egypt; National Institutes of Health, UNITED STATES

## Abstract

In liver fibrosis, conversion of fibroblasts to profibrogenic myofibroblasts significantly drives the development of the disease. A crucial role of cyclic adenosine monophosphate (cAMP) in regulation of fibroblast function has been reported. Increase in cAMP levels has been found to decrease fibroblast proliferation, inhibit their conversion to myofibroblast, and stimulate their death. cAMP is generated by adenyl cyclase (AC), and degraded by cyclic nucleotide phosphodiesterase (PDE). In this study, the antifibrotic effect of a PDE inhibitor, cilostazol (Cilo), on a rat model of liver fibrosis induced by thioacetamide (TAA) was investigated. Four groups of rats were used; the first group received the vehicles and served as the normal control group, while liver fibrosis was induced in the other groups using (TAA, 200 mg/kg/biweekly for 8 successive weeks, *ip*). The last two groups were treated with Cilo (50 and 100 mg/kg/day, *po*, respectively). Induction of liver fibrosis in TAA-treated rats was observed as evidenced by the biochemical and histopathological findings. On the other hand, a potent antifibrotic effect was observed in the groups treated with Cilo, with preference to the higher dose. In these groups, a significant increase in the liver content of cAMP was demonstrated that was accompanied by reduction in the hepatic expression of key fibrogenic cytokines, growth factors, and inflammatory biomarkers, including interleukin-6, tumor necrosis factor-alpha, nuclear factor kappa B, and transforming growth factor-beta as compared to TAA group. Moreover, amelioration of TAA-induced oxidative stress and apoptosis in the liver has been observed. These findings reveal the antifibrotic effect of Cilo against TAA-induced liver fibrosis in rats, and suggest regulation of cAMP pathway, together with the modulation of oxidative stress, inflammation, and apoptosis as mechanistic cassette underlines this effect.

## Introduction

Liver fibrosis is a major health problem that threatens life and results in a significant percent in morbidity and mortality [1]. It is a reversible wound-healing response that occurs as a result of acute or chronic liver injury. The pathogenesis of liver injury is complex and it is characterized by the deposition of extracellular matrix (ECM) [2–4]. All liver cell types has their own role in the development of liver fibrosis, and it is now believed that there is a cross talk between cells of different types in the process of fibrosis through specific mediators released as interleukins, growth factors and reactive oxygen species (ROS) [5]. Many strategies have been made to attenuate liver fibrosis by inhibiting pathways or inhibiting certain molecular targets involved in the development of liver fibrosis [6].

In contrast, some protective molecular pathways become repressed during the course of liver injury. One of these important including signaling pathways is the cyclic adenosine monophosphate (cAMP) pathway [7]. cAMP is a second messenger that plays a central role in cellular responses to neurotransmitters and hormones [8]. It regulates various cellular functions including inflammation and cell differentiation by affecting gene/protein expression and function [9]. Increasing cAMP levels has been found to inhibit the conversion of resting fibroblasts/fibroblast-like cells (such as hepatic stellate cells, HSC) to profibrogenic myofibroblasts after cell injury [10], decrease their proliferation [11], stimulate their death [12], and inhibit ECM protein synthesis [13, 14]. Hence, cAMP pathway is a potential target to blunt fibrosis [6].

cAMP is generated by adenyl cyclase (AC) in response to activation of G-protein-coupled receptors, and degraded by cyclic nucleotide phosphodiesterase (PDE) into adenosine monophosphate; thus, both AC and PDE regulate the intracellular level of cAMP [15]. Cilostazol (Cilo) is a selective type-3 PDE inhibitor that is used to inhibit platelet aggregation by increasing the intracellular cAMP. Besides, Cilo showed other cAMP pathway-dependent and -independent pharmacological effects including anti-inflammatory [16, 17], antioxidant [18], and anti-apoptotic actions [19, 20]. A beneficial effect of Cilo against some rodents models of liver injury induced by ethanol [19], galactosamine [21], ischemia reperfusion [22], and carbon tetrachloride (CCl_4_) [23] has been demonstrated. However, its potential hepatoprotective effect against thioacetamide (TAA)-induced hepatic fibrogenesis in rats has not been evaluated, which is the aim of the current experiment, along with the study of the possible mechanistic scenario.

## Materials and methods

### Drugs and chemicals

TAA was purchased from Sigma-Aldrich Co. (USA) and was dissolved in 0.9% w/v saline solution for intraperitoneal (*ip*) injection. Cilo was purchased from Otsuka Pharmaceuticals (UK), and used orally (*po*). All other chemical reagents were purchased from Sigma-Aldrich chemical Co. (USA).

### Experimental animals

Animals used were adult male Wister rats, weighing 150–200 g each, purchased from the Animal House at the National Research Centre (NRC, Egypt). Rats were maintained under standard conditions of temperature (25 °C) with a 12 h (light)– 12 h (dark) cycle, and were allowed free access to standard laboratory food and water. This study has been approved by the ethics committees of the Committee of Animal Care and Use of NRC (Egypt). All procedures and experiments were performed according to the protocol approved by them, and the animals were treated according to the national and international ethics guidelines. The earliest scientifically justified endpoint was used in this study to prevent pain or distress in the experimental animals. Rats were sacrificed by cervical dislocation under ether anesthesia.

### Induction of liver fibrosis and experimental design

Rats were randomly allocated into four groups (8 animals per group), and treated for eight consecutive weeks according to the following scheme:

1. The normal control group, where rats received saline *ip* twice weekly, and the vehicle orally once daily.
2. The fibrotic control (TAA group), where rats received TAA (200 mg/kg, *ip*) twice weekly to provoke liver fibrosis [24], and received the vehicle orally once daily.
3. TAA-Cilo50 group, where rats received Cilo (50 mg/kg/day, *po*) [25] for eight consecutive weeks, together with injection of TAA twice weekly.
4. TAA-Cilo100 group, where rats received Cilo (100 mg/kg/day, *po*) [25] for eight consecutive weeks, together with injection of TAA twice weekly.

Twenty-four hours following the last drug administration, blood samples were withdrawn from the retro-orbital plexus of the rats under light ether anesthesia. Then, rats were sacrificed by cervical dislocation under the same anesthesia for collection of liver samples. A weighed part of the liver of each animal was rapidly dissected out, washed and homogenized using phosphate-buffered saline (PBS, 50 mM potassium phosphate, pH 7.5) at 4°C to produce a 20% homogenate. Liver homogenates were kept at -80°C till time of analysis. Another part of liver tissues were kept in 10% formalin-saline for histopathological examination.

### Assessment of liver functions biomarkers

Levels of serum alanine aminotransferase (ALT) and aspartate aminotransferase (AST) were determined colorimetrically using commercially available kits (Biodiagnostic, Egypt) [26].

### Assessment of oxidative stress markers

Reduced glutathione (GSH) was determined in the liver homogenate with Ellman’s reagent according to a previously described method [27]. In addition, the products of lipid peroxidation (mainly malondialdehyde, MDA) were determined as thiobarbituric acid-reactive substances [28].

### Enzyme-linked immunosorbent assay (ELISA) of some biomarkers in liver homogenate

Liver homogenates were assayed for cAMP using specific Rat ELISA kit (R&D Systems, USA; Catalogs Number: KGE012B).

Tumor necrosis factor (TNF-α), interleukin-6 (IL-6), and nuclear factor-kappa B (NF-κB) as important signals in liver injury [29], were also assayed, as well as transforming growth factor-beta (TGF-β) as a fibrogenesis-driving cytokine [30], using specific Rat ELISA kits (SunLong Biotech Co., LTD, China; Catalogs Number: SL0722Ra, SL0411Ra, SL0537Ra, and SL0705Ra, respectively).

As caspase-3 is involved in the apoptosis of hepatocytes during liver injury [31], CUSABIO Rat Casp-3 ELISA Kit (USA; Catalog Number: CSB-E08857r) was used to measure the level of caspase-3 in liver homogenate. Moreover, evaluation of α-smooth muscle actin (α-SMA) and type I collagen (COL-1) as classic liver fibrosis markers was also performed using specific Rat ELISA kits (CUSABIO, USA; Catalogs Number: CSB-E 14407r and CSB-E10134r, respectively).

### Histopathological examination

For histopathological examination, morphometry and fibrosis assessment, liver tissues were immediately fixed to 10% formal saline for paraffin embedding, sectioning, and staining with Hematoxylin & Eosin (H&E) and Masson trichrome stains. The Masson trichrome specifically stain the collagen to give blue color staining of the fibrous tissue. Quantitative analysis was done in the Pathology Department of the NRC, using the Leica Qwin 500 Image Analyzer (LEICA Imaging Systems Ltd, Cambridge, England).

Ten random fields were examined for estimation of fibrous tissue percent, portal tract area (μm^2^), inflammatory cell infiltration, and hepatocytic degeneration. The mean from these ten fields was calculated which represent the histopathological condition of the liver tissue.

### Statistical analysis

All the values are presented as means ± standard error of the means (SE), n = 8. Comparisons between different groups were carried out using one-way analysis of variance (ANOVA) followed by Tukey-Kramer test for multiple comparisons. Graphpad Prism software, version 5 (USA) was used to carry out these statistical tests. The difference was considered significant when *p* ˂ 0.05.

## Results

### Effect of Cilo on the serum AST and ALT levels in TAA-induced liver fibrosis in rats

**Fig 1:**
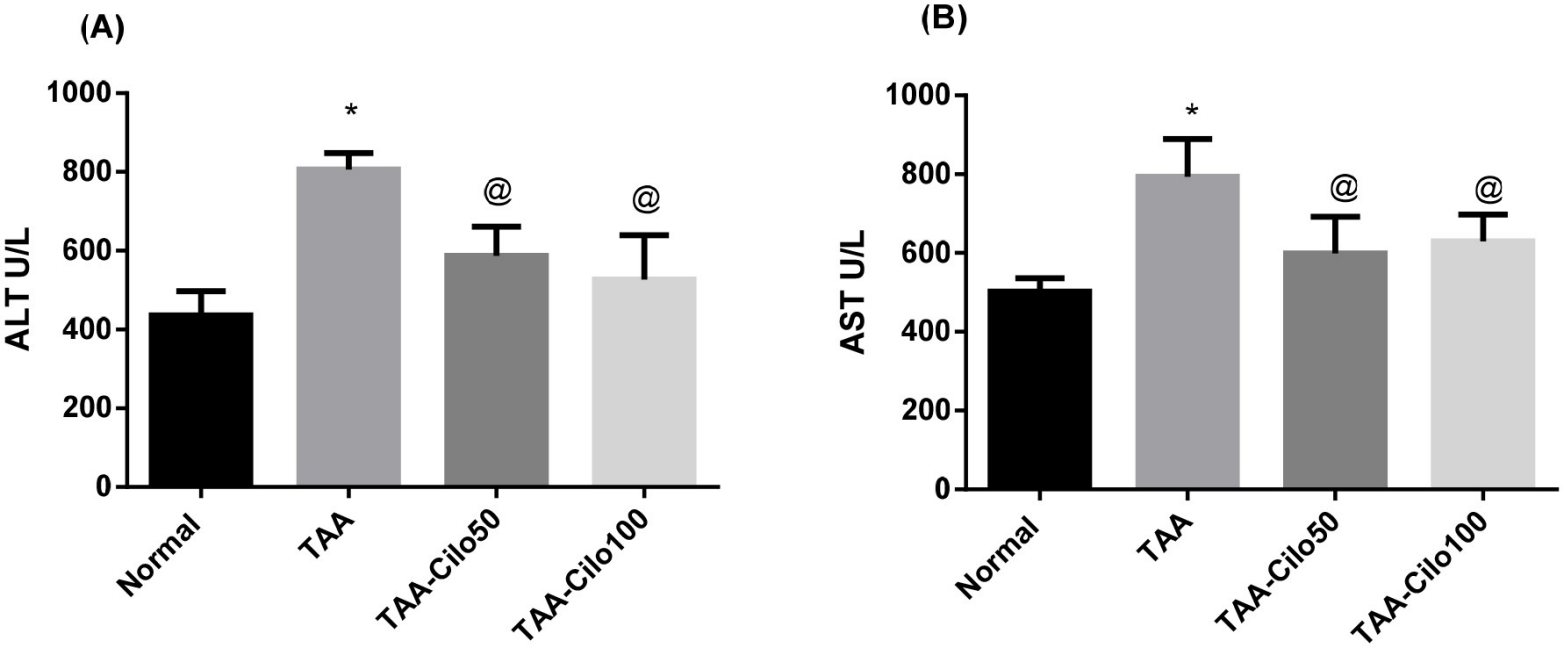
Effect of cilostazol on the serum ALT (A), AST (B) levels in rats with TAA-induced liver fibrosis. Normal, rats treated with vehicles; TAA, rats treated with thioacetamide (200 mg/kg/biweekly for 8 weeks, *ip*); TAA-Clio, rats treated with thioacetamide and cilostazol (50 or 100 mg/kg/day for 8 weeks, *po*); ALT, alanine aminotransferase; AST, aspartate aminotransferase. Data presented as mean ± S.E.; n = 8. * Significantly different from Normal group at *p*˂0.05. @ Significantly different from TAA group at *p*˂0.05.

Injection of TAA resulted in a considerable elevation in serum ALT and AST levels as compared to normal group values (1.85 and 1.57 fold, respectively). Both Clio-treated groups had a significant decrease in serum ALT and AST when compared to TAA control group. Cilo 50 and 100 mg/kg depleted the raised ALT levels by 27%, and 35%, respectively, as compared to TAA control group. Moreover, cilostazol 50 and 100 mg/kg depleted the raised AST levels by 24%, and 21%, respectively as compared to TAA control group (Fig 1).

### Effect of Cilo on the liver contents of oxidative stress markers in TAA-induced fibrosis in rats

**Fig 2:**
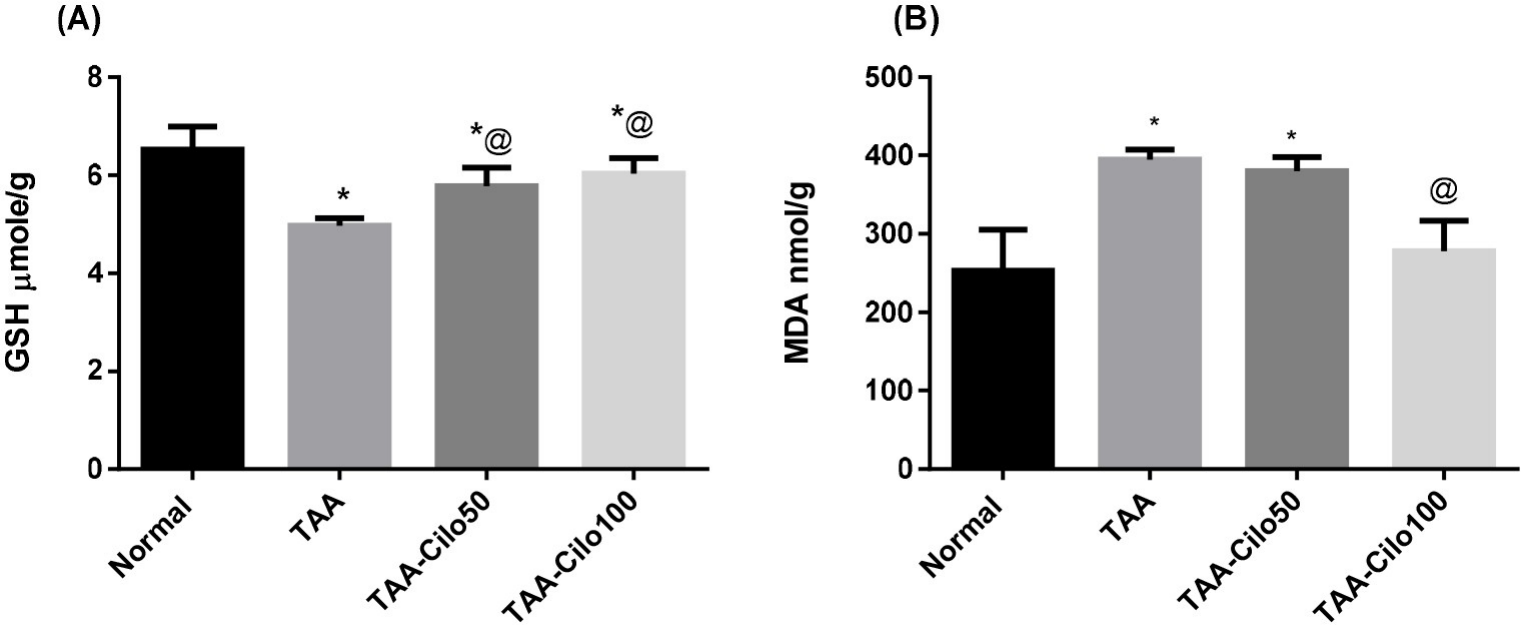
Effect of cilostazol on liver GSH (A) and MDA (B) in TAA-induced fibrosis in rats. Normal, rats treated with vehicles; TAA, rats treated with thioacetamide (200 mg/kg/biweekly for 8 weeks, *ip*); TAA-Clio, rats treated with thioacetamide and cilostazol (50 or 100 mg/kg/day for 8 weeks, *po*); GSH: reduced glutathione, MDA, malondialdehyde. Data presented as mean ± S.E.; n = 8. * Significantly different from Normal group at *p*˂0.05. @ Significantly different from TAA group at *p*˂0.05.

Injection of TAA resulted in a significant depletion in reduced glutathione (GSH) levels (24%) (Fig 2A) as well as a significant elevation in MDA values (50%) as compared to normal group values (Fig 2B). On the other hand, Cilo 50 and 100 mg/kg raised the depleted GSH levels by 16% and 21%, respectively, while decreased the raised MDA levels by 4% and 30%, respectively, as compared to TAA control group.

### Effect of Cilo on the liver content of cAMP in TAA-induced fibrosis in rats

**Fig 3:**
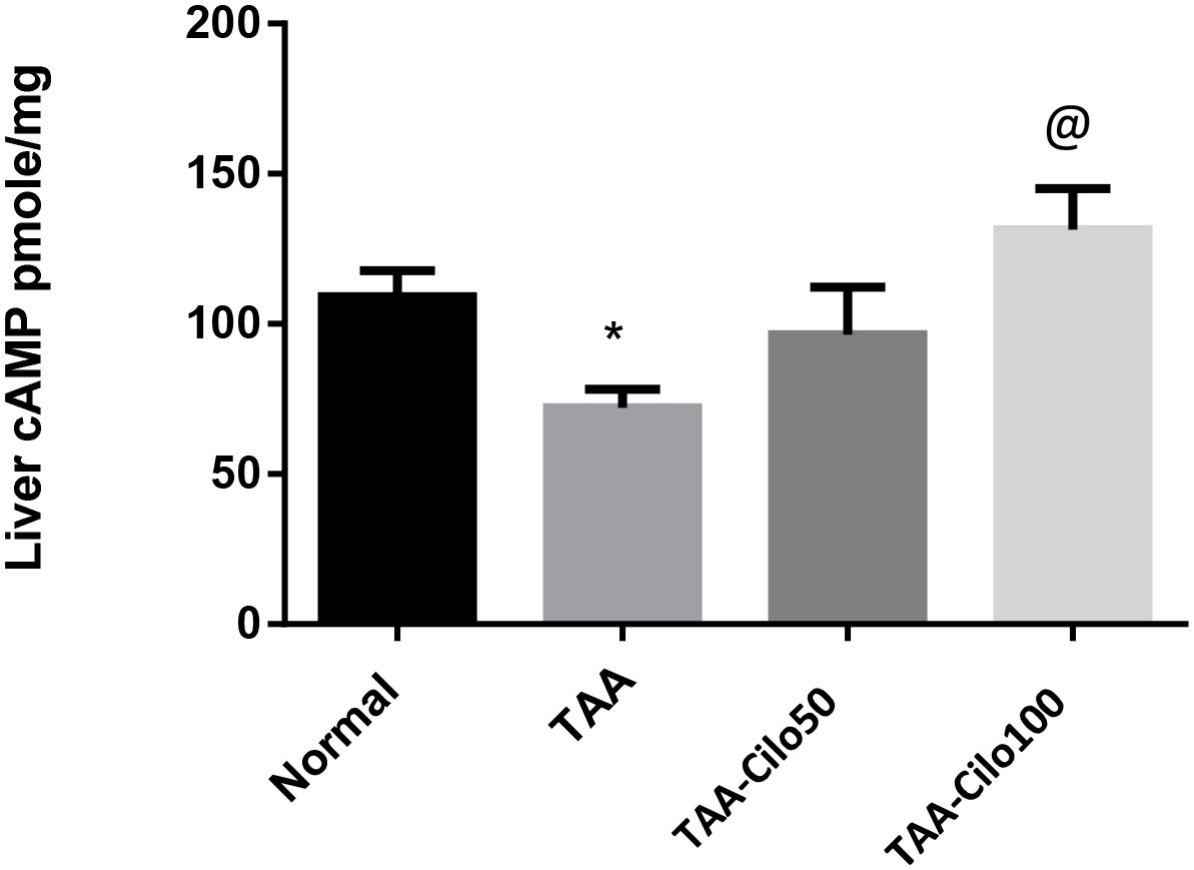
Effect of cilostazol on liver content of cAMP in TAA-induced fibrosis in rats. Normal, rats treated with vehicles; TAA, rats treated with thioacetamide (200 mg/kg/biweekly for 8 weeks, *ip*); TAA-Clio, rats treated with thioacetamide and cilostazol (50 or 100 mg/kg/day for 8 weeks, *po*); cAMP: cyclic adenosine monophosphate. Data presented as mean ± S.E.; n = 8. * Significantly different from Normal group at *p*˂0.05. @ Significantly different from TAA group at *p*˂0.05.

A significant reduction of the normal hepatic content of cAMP was observed in the rats with TAA-induced liver fibrosis. However, a dose-dependent increase of this content was observed in the groups treated with Cilo, 50 and 100 mg/kg (Fig 3).

### Effect of Cilo on the hepatic contents of some cytokines and chemokines in TAA-induced fibrosis in rats

Injection of TAA resulted in a significant elevations in liver TNF-α, IL-6, NFkβ and TGF-β (2.14, 2.1, 2.13 and 2.3 fold respectively) as compared to normal group values.

**Fig 4:**
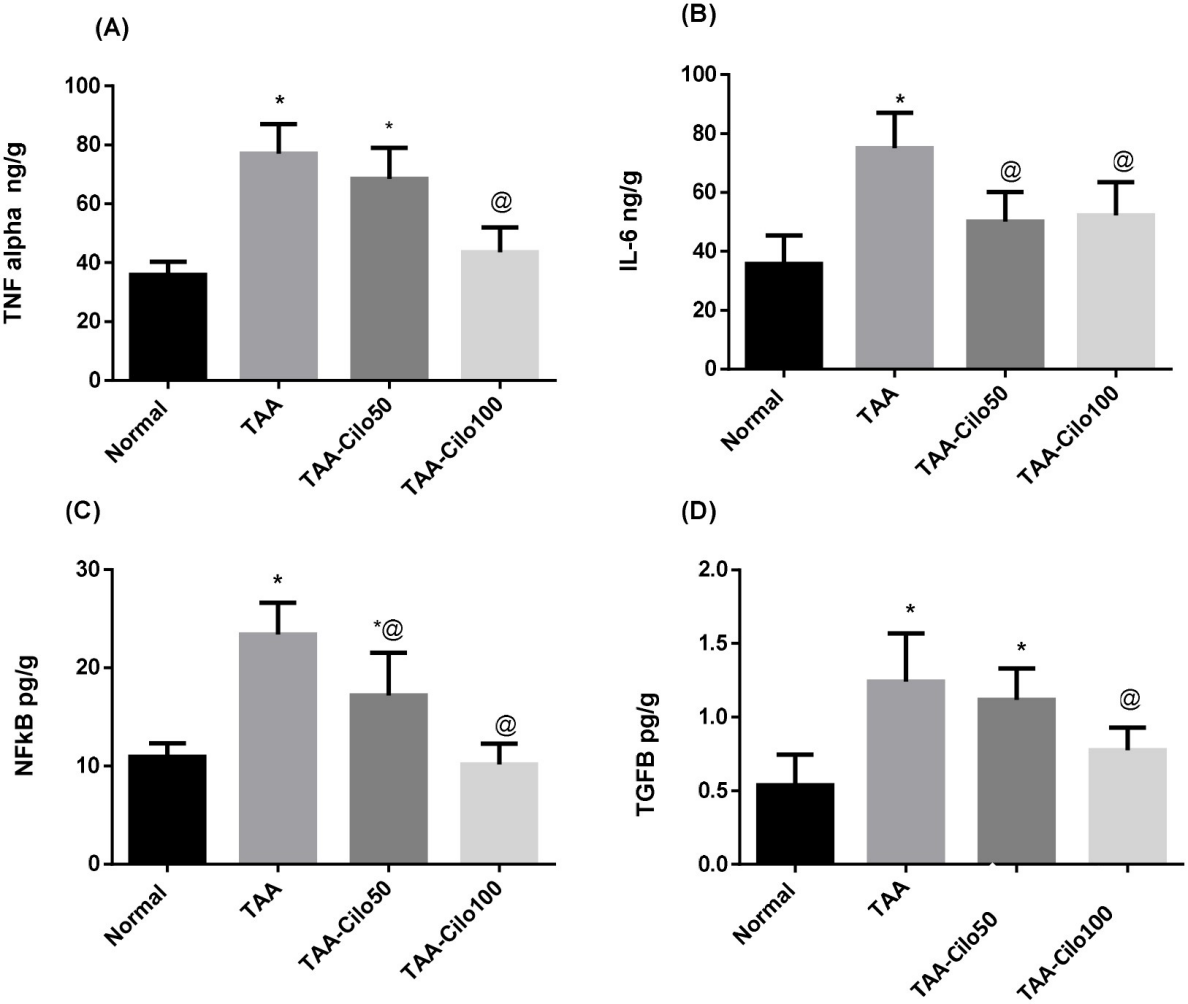
Effect of cilostazol on liver TNF-α (A), IL-6 (B), NFκβ (C) and TGFβ (D) in TAA-induced fibrosis in rats. Normal, rats treated with vehicles; TAA, rats treated with thioacetamide (200 mg/kg/biweekly for 8 weeks, *ip*); TAA-Clio, rats treated with thioacetamide and cilostazol (50 or 100 mg/kg/day for 8 weeks, *po*); TNF-α: tumor necrosis factor α; IL-6, interleukin-6; NF-κB, nuclear factor-kappa B; TGF-β, transforming growth factor-beta. Data presented as mean ± S.E.; n = 8. * Significantly different from Normal group at *p*˂0.05. @ Significantly different from TAA group at *p*˂0.05.

Only Cilo 100 mg/kg significantly decreased the raised TNF-α and TGF-β hepatic levels as compared to TAA control group. On the other hand, both doses of cilo, 50 and 100 mg/kg, significantly decreased the raised IL-6 levels by 33% and 31%, respectively, as well as NF-kβ levels by 27 and 56%, respectively, comparing to TAA control group (Fig 4).

### Effect of Cilo on caspase-3 in TAA-induced fibrosis in rats

**Fig 5:**
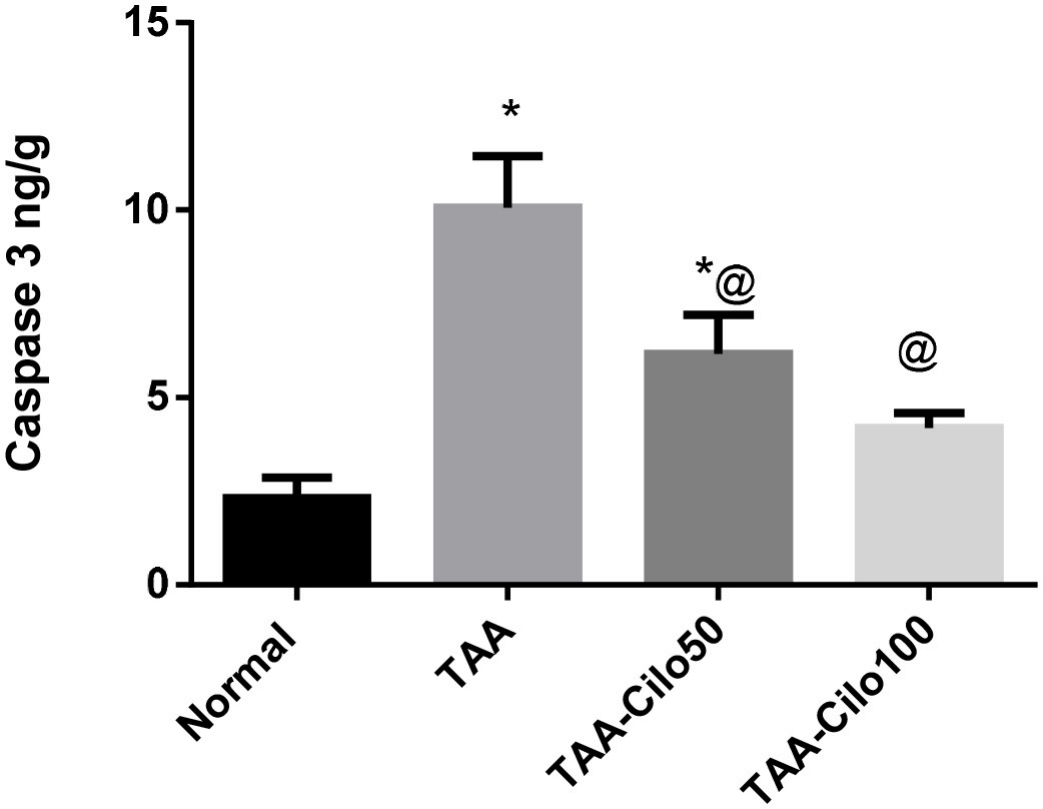
Effect of cilostazol on liver caspase-3 in TAA-induced fibrosis in rats. Normal, rats treated with vehicles; TAA, rats treated with thioacetamide (200 mg/kg/biweekly for 8 weeks, *ip*); TAA-Clio, rats treated with thioacetamide and cilostazol (50 or 100 mg/kg/day for 8 weeks, *po*); Caspase 3. Data presented as mean ± S.E.; n = 8. * Significantly different from Normal group at *p*˂0.05. @ Significantly different from TAA group at *p*˂0.05.

Induction of liver fibrosis in rats using TAA resulted in a significant elevation in hepatic caspase-3 (4.7-fold) as compared to normal group value (Fig 5). Treatment of rats with Cilo, 50 and 100 mg/kg, significantly decreased the raised hepatic caspase-3 levels by 44% and 62%, respectively, as compared to TAA group.

### Effect of Cilo on α-SMA and COL-1 in TAA-induced fibrosis in rats

**Fig 6:**
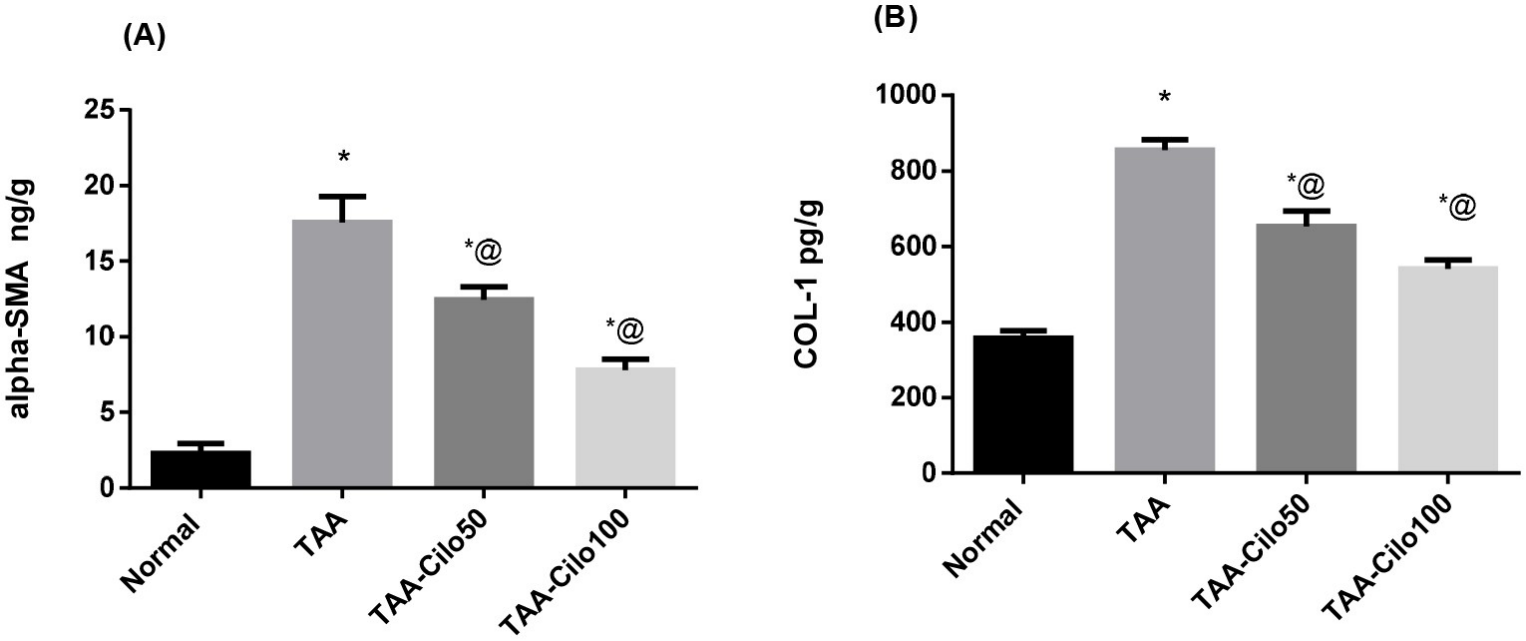
Effect of cilostazol on liver α-SMA (A) and COL-1 (B) in TAA-induced fibrosis in rats. Normal, rats treated with vehicles; TAA, rats treated with thioacetamide (200 mg/kg/biweekly for 8 weeks, *ip*); TAA-Clio, rats treated with thioacetamide and cilostazol (50 or 100 mg/kg/day for 8 weeks, *po*); α-SMA, α-smooth muscle actin; COL-1, type I collagen. Data presented as mean ± S.E.; n = 8. * Significantly different from Normal group at *p*˂0.05. @ Significantly different from TAA group at *p*˂0.05.

Induction of liver fibrosis in rats using TAA resulted in a significant elevation in hepatic α-SMA and COL-1 as compared to the normal group (Fig 6). Treatment of rats with Cilo, 50 and 100 mg/kg, significantly decreased the raised hepatic α-SMA and COL-1 levels comparing with TAA group.

### Effect of Cilo on the liver histopathological findings in TAA-induced fibrosis in rats

**Fig 7:**
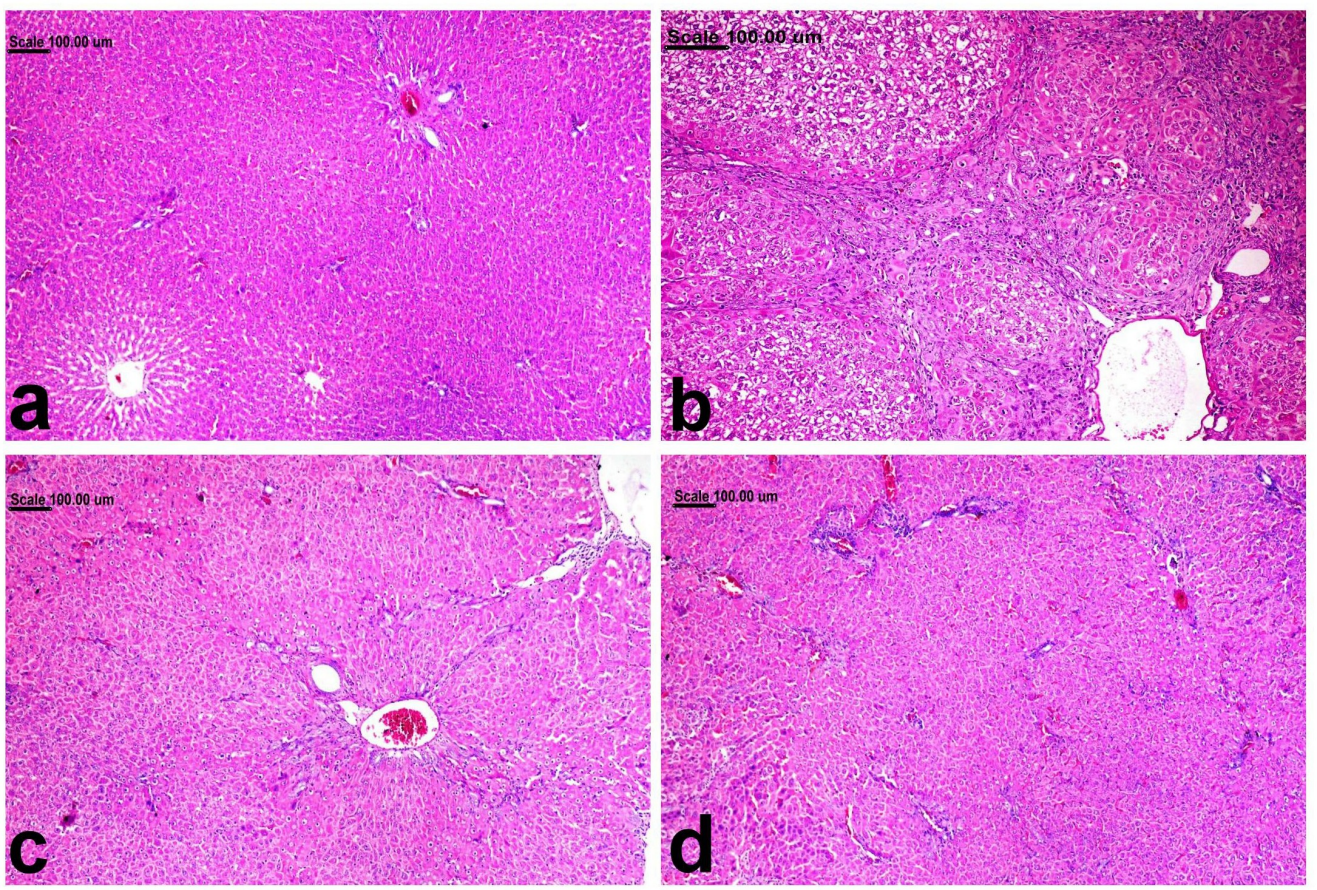
liver tissue, stained with H&E, of (a) normal rats showing normal hepatic parenchyma with normal hepatocytes, (b) TAA-treated group showing disrupted hepatic parenchyma, with massive fibrous tissue proliferation extending from the portal area, associated with ballooning degeneration of hepatocytes in addition to apoptosis, (c) TAA-Cilo50-treated group showing diminished fibrosis with minimal inflammatory infiltrates, and (d) TAA-Cilo100-treated group showing normal preserved parenchyma. (H&E, 20X). Normal, rats treated with vehicles; TAA, rats treated with thioacetamide (200 mg/kg/biweekly for 8 weeks, *ip*); TAA-Clio, rats treated with thioacetamide and cilostazol (50 or 100 mg/kg/day for 8 weeks, *po*).

**Fig 8:**
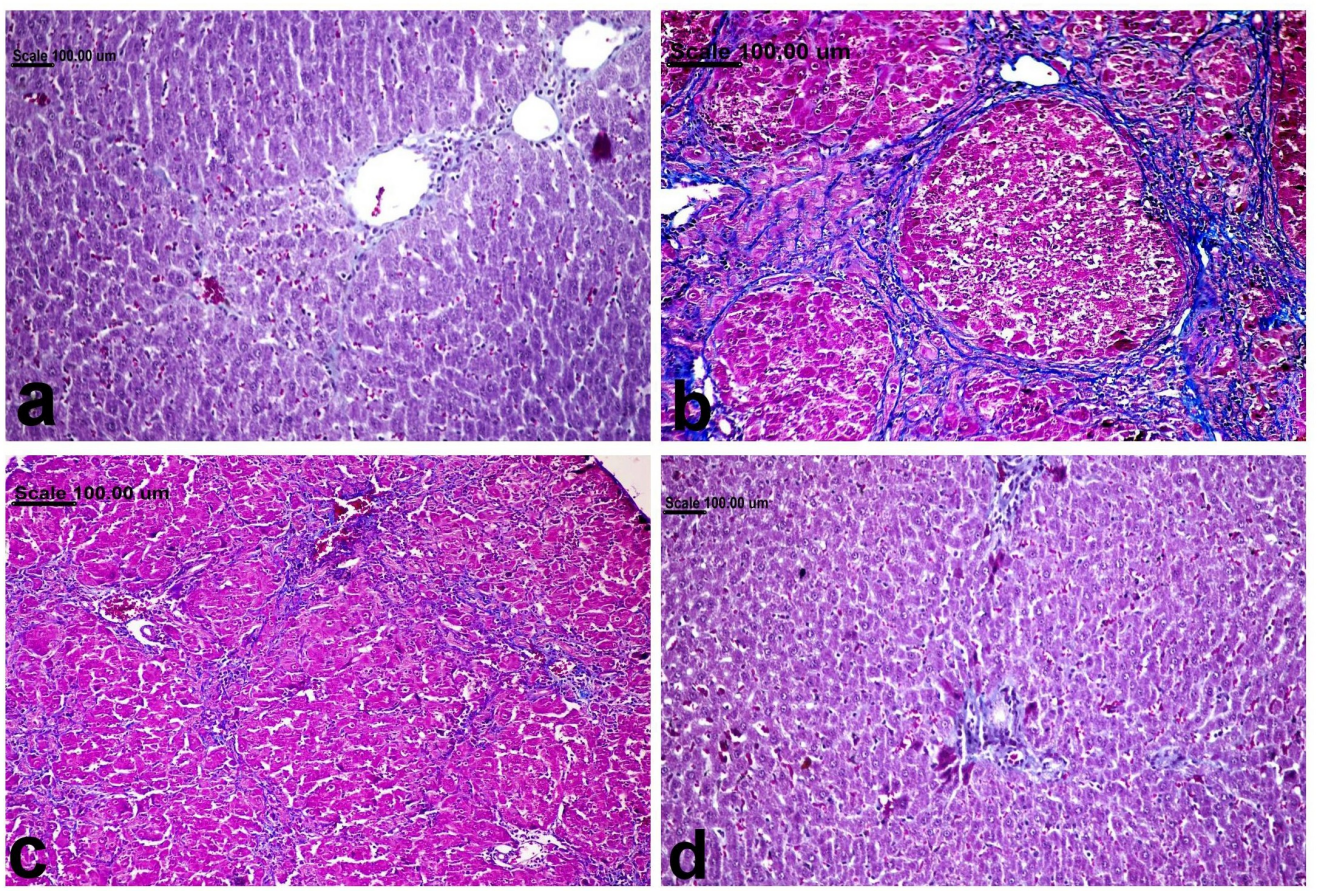
liver tissue, stained with Masson´s trichrome staine, of (a) normal rats showing scant faint blue-stained fibrous in the portal area, (b) TAA-treated group showing strong blue-stained collagen fiber, (c) TAA-Cilo50-treated group showing diminished blue-stained collagen fibers, and (d) TAA-Cilo100-treated group showing scarce blue-stained fibrous tissue confined to the portal area. (Masson´s trichrome staine, 20X). Normal, rats treated with vehicles; TAA, rats treated with thioacetamide (200 mg/kg/biweekly for 8 weeks, *ip*); TAA-Clio, rats treated with thioacetamide and cilostazol (50 or 100 mg/kg/day for 8 weeks, *po*).

Liver of normal rats revealed normal hepatic parenchyma with normal hepatocytes (Fig 7a) and scant faint blue-stained fibrous tissue demonstrated in the portal area (Fig 8a). On the contrary, disrupted hepatic parenchyma, with massive fibrous tissue proliferation extending from the portal area, associated with ballooning degeneration of hepatocytes in addition to apoptosis, was demonstrated in TAA-treated group (Fig 7b). Atypical biliary epithelial hyperplasia associated with leukocytic cell infiltration was characteristically demonstrated in this group. The collagen fibers were strongly blue in Masson´s trichrome stained sections (Fig 8b). Diminution of fibrosis with minimal inflammatory infiltrates were demonstrated in TAA-Cilo50-treated group (Figs 7c and 8c). Interestingly, pronounced amelioration was demonstrated in TAA-Cilo100-treated group, in which the hepatic parenchyma in most examined sections appeared normal with scarce fibrous tissue confined to the portal area (Figs 7d and 8d) (Table 1).

**Table 1:** Histopathological parameters of the liver in the studied groups.

	Fibrous tissue%	Portal tract area (μm^2^)	Inflammatory cell infiltrate	Hepatocyte degeneration
**Normal**	2.4	180.2	---	---
**TAA**	7.6	894.3	++	+ steatosis
**TAA-Cilo50**	4.5	335.4	+	+ hydropic degeneration
**TAA-Cilo100**	4.3	319.4	-	-

Normal, rats treated with vehicles; TAA, rats treated with thioacetamide (200 mg/kg/biweekly for 8 weeks, *ip*); TAA-Clio, rats treated with thioacetamide and cilostazol (50 or 100 mg/kg/day for 8 weeks, *po*). Values represent the means in ten fields.

## Discussion

The current study has targeted evaluation of Cilo as a therapeutic alternative to liver fibrosis and analyzing the mechanism by which it acts to attenuate liver fibrosis elicited in rats by TAA. Induction of liver fibrosis by TAA is occurred as a result of its biotransformation, by CYP2E1 enzymes in the liver cells’ microsomes, to a very reactive intermediate known as TAA sulphur dioxide through oxidation [32]. This results in variable grades of hepatotoxicity including nodular cirrhosis, proliferation of hepatic cells, and necrosis of parenchyma cells [33]. TA is a common hepatotoxicant model to study liver injury due to its outstanding solubility in the water, and a prolonged injury and recovery pattern giving significant time to study mechanisms [34, 35]. Unlike CCl4, TA toxicity does not depend on induction of oxidative stress only [36]. Therefore, using TAA-induced liver fibrosis model allows assessing different pathophysiological factors, parameters and mechanisms that may underline the progression of liver diseases rather than oxidative stress only. This adds an advantage to the TAA-hepatotoxic model by its potential to simulate some clinical conditions where many precipitating factors are involved in the development of liver fibrosis, and different mechanisms underline its progression [2–4]. Moreover, because of its limited use as a model toxicant comparing to other experimental animal models, especially in the chronic studies, the exact mechanism of TA-induced necrotic cell death is not clear. Thus, using it in more studies investigating different pathways are required and would add to the knowledge.

In the present study, TAA-induced liver damage in rats was evidenced, biochemically, by the alterations in serum levels of ALT and AST, as well as hepatic fibrosis biomarkers in TAA-induced group, and confirmed by the histopathological findings. Moreover, induction of oxidative stress state in the current TAA-treated group has been proved by the decline in the normal liver GSH content as well as an elevation in MDA content. These results are in accordance with previous studies showed that TAA, by its oxidative stress capacity that exceeds the capacity of body’s antioxidative and protective mechanisms [37], damages liver cell membranes, resulting in the leakage of the cytoplasmic liver enzymes ALT and AST into blood stream in amounts related to the extent of liver damage [38–40].

Administration of Cilo in the current study significantly improved the liver function, oxidative stress, and liver fibrosis biomarkers, together with the histopathological findings, when compared to the control TAA group; this was in preference to the higher dose (100 mg/kg). These findings indicate the beneficial effects of Cilo in the healing and regeneration of the hepatocytes, and show its antioxidant potential [18]. Same effects of Cilo have been shown before when other research groups used other rodent’s models of liver fibrosis induced by ethanol and CCl_4_ [19, 23]. Other PDE inhibitor, pentoxifylline, has shown similar results against TAA-induced model [41].

In the current TAA-induced liver fibrosis model, reduction of the hepatic cAMP content was observed. This support the data that cAMP pathway is considered as protective signaling pathway against liver fibrosis that become repressed during liver injury [7]. In line with that, the increased level of cAMP observed in the liver of the rats treated with the PDE inhibitor, Cilo, justifies its hepatoprotective effects.

The liver is considered as a main organ controlling cytokines activity and its production depending on the initial early pro-inflammatory cytokines released from macrophages [42, 43]. TAA, as a hepatotoxin, induces pro-inflammatory and inflammatory cytokines and mediators by macrophages (Kupffer cells) such as TNF-α, IL-6, NF-κβ and TGFβ that play important roles in hepatic inflammation [44, 45]. The findings of our study are in line with these previous observations. NF-κB plays an important role in the expression of many pro-inflammatory genes [46], and its induction has been found to play a crucial role in TAA-induced liver fibrosis [47, 48]. After activation with pro-inflammatory cytokines, NF-ĸB acts as the driving force of fibrosis. It activates TGFβ, which is considered as the key mediator in fibrogenesis as it potentiates the conversion of HSCs into myofibroblasts that stimulates the synthesis of ECM proteins, and hinders its degradation [49]. An increase in the relative genetic expression of TGFβ due to TAA administration has been reported [50, 51].

Many studies have shown that HSCs activation can be attenuated by blocking the NFκβ signaling pathway [52, 53]. In our study, Cilo, in a dose-dependent manner, significantly reduced the hepatic proinflammatory cytokines, TNF-α and IL-6, contents, as well as NFκβ and TGFβ1 levels when compared to TAA-induced group. These findings suggest that one of the possible mechanisms involved in the antifibrotic effect of Cilo might be through its previously reported anti-inflammatory action [16, 17] with suppression of proinflamatory and fibrogenic cytokines.

Moreover, elevation of caspase-3, as a marker of apoptosis, was odserved in the liver of TAA-treated rats. Fourteen caspases have been involved in the apoptotic pathway cascade [54]. Among these, caspase-3 is considered to be a major execution protease. Caspase-3 is a sensitive marker reflecting liver damage and is associated with liver fibrosis as well [55]. It is suggested that the inflammation that occurred in the liver cells elicited by TAA lead in the later stages to apoptosis of hepatocytes reflected by the elevated level of caspase-3 in the TAA-induced rats. Cilo in both doses significantly depleted caspase-3 level elevation, indication a protective and anti-apoptotic effect of Cilo. This observation is in agreement with other data reported the anti-apoptotic effect of Cilo [56, 57].

## Conclusion

These findings revealed that the PDE inhibitor Cilo showed hepatoprotective effects against TAA-induced liver fibrosis in rats. It modulated many process in the liver, including the oxidative stress, cAMP pathway, inflammation, ECM and collagen deposition, as well as apoptosis. The study proposes that these effects worked together to reduce fibrogenesis, and suggest Cilo as a promising antifibrotic agent against liver fibrosis. More comprehensive mechanistic studies are recommended to bring to light the antifibrotic role of Cilo in liver injury.

## Supporting information

S1 File Raw data-PLOS one-Elawdan et al.docx. (DOCX)
