# Supplementary material for: Regression of fibrosis by cilostazol in a rat model of thioacetamide-induced liver fibrosis: Up regulation of hepatic cAMP, and modulation of inflammatory, oxidative stress and apoptotic biomarkers
Source: PLoS One. 2019 May 8;14(5):e0216301. doi: 10.1371/journal.pone.0216301 (PMC6505801; doi:10.1371/journal.pone.0216301)
Supplement: S1 File — (DOCX) [file pone.0216301.s001.docx]

| Group | No. | Caspase 3 (ng/g) | Alpha-SMA (ng/g) | COL-I (pg/g) | TGF B (pg/g) | MDA (nM/g) | IL-6 (ng/g) | TNF alpha (ng/g) | NFKb (pg/g) | GSH (μM/g) | ALT (U/L) | AST (U/L) | liver cAMP (pMol/mg) |
| --- | --- | --- | --- | --- | --- | --- | --- | --- | --- | --- | --- | --- | --- |
|  |  |  |  |  |  |  |  |  |  |  |  |  |  |
| Normal | 1 | 1.98 | 1.25 | 365.45 | 0.43 | 481.67 | 26.92 | 26.75 | 8.96 | 6.40 | 458.00 | 542.89 | 89.40 |
|  | 2 | 2.11 | 1.65 | 334.98 | 0.50 | 444.62 | 48.64 | 25.75 | 20.02 | 6.08 | 483.00 | 470.99 | 137.50 |
|  | 3 | 3.11 | 4.85 | 370.38 | 0.35 | 347.44 | 22.59 | 29.00 | 11.03 | 7.19 | 557.00 | 516.90 | 112.00 |
|  | 4 | 2.85 | 0.89 | 384.16 | 0.63 | 175.26 | 55.35 | 35.75 | 8.18 | 6.13 | 689.00 | 478.98 | 126.90 |
|  | 5 | 1.87 | 3.28 | 289.48 | 0.92 | 164.50 | 24.49 | 54.16 | 7.80 | 6.14 | 357.18 | 538.86 | 88.90 |
|  | 6 | 2.43 | 0.98 | 365.21 | 0.79 | 255.64 | 49.03 | 35.37 | 16.59 | 8.05 | 269.30 | 458.79 | 125.00 |
|  | 7 | 1.88 | 2.78 | 384.09 | 0.24 | 148.79 | 43.00 | 38.66 | 8.38 | 6.02 | 435.13 | 534.97 | 89.00 |
|  | 8 | 2.30 | 3.67 | 365.43 | 0.58 | 138.00 | 34.50 | 42.51 | 9.46 | 6.15 | 259.38 | 476.84 | 116.50 |
| TAA | 1 | 12.38 | 21.28 | 926.64 | 0.71 | 412.82 | 78.23 | 104.15 | 18.48 | 4.90 | 623.00 | 793.69 | 76.75 |
|  | 2 | 10.75 | 19.65 | 893.76 | 1.29 | 382.05 | 122.91 | 56.75 | 25.53 | 5.10 | 837.20 | 838.90 | 87.25 |
|  | 3 | 11.50 | 16.98 | 867.94 | 1.72 | 394.23 | 67.99 | 71.78 | 13.73 | 5.17 | 947.00 | 844.64 | 69.33 |
|  | 4 | 9.04 | 14.94 | 836.69 | 1.34 | 390.38 | 51.18 | 66.95 | 26.25 | 4.90 | 863.00 | 754.62 | 64.50 |
|  | 5 | 10.03 | 18.92 | 866.60 | 1.78 | 435.26 | 57.74 | 113.75 | 33.35 | 4.82 | 781.46 | 676.49 | 59.50 |
|  | 6 | 10.20 | 16.88 | 789.90 | 1.23 | 444.58 | 67.89 | 73.87 | 24.47 | 4.80 | 834.20 | 955.49 | 76.70 |
|  | 7 | 12.24 | 19.34 | 779.81 | 0.84 | 362.15 | 78.69 | 70.65 | 26.46 | 5.25 | 826.67 | 794.33 | 80.60 |
|  | 8 | 12.12 | 12.35 | 864.69 | 1.25 | 337.48 | 80.23 | 66.72 | 19.35 | 4.88 | 769.42 | 689.04 | 75.50 |
| TAA-Cilo50 | 1 | 7.40 | 12.15 | 667.30 | 1.45 | 382.05 | 69.34 | 56.25 | 8.49 | 5.48 | 794.78 | 578.90 | 124.90 |
|  | 2 | 6.35 | 13.75 | 637.49 | 0.89 | 448.72 | 36.92 | 81.25 | 10.85 | 5.77 | 659.93 | 454.75 | 64.45 |
|  | 3 | 5.38 | 15.35 | 682.20 | 0.99 | 403.85 | 72.54 | 54.12 | 29.49 | 5.98 | 367.48 | 799.66 | 81.90 |
|  | 4 | 4.90 | 14.98 | 672.06 | 1.12 | 293.15 | 59.77 | 60.25 | 9.53 | 6.88 | 779.84 | 568.71 | 75.25 |
|  | 5 | 5.24 | 8.72 | 564.20 | 0.72 | 361.50 | 28.54 | 103.47 | 10.44 | 5.89 | 473.56 | 767.60 | 71.38 |
|  | 6 | 6.57 | 14.73 | 667.29 | 1.12 | 426.40 | 52.40 | 110.35 | 8.97 | 6.49 | 614.57 | 658.70 | 123.70 |
|  | 7 | 7.03 | 9.75 | 579.31 | 0.96 | 376.37 | 38.64 | 73.45 | 31.65 | 5.07 | 582.44 | 583.60 | 96.50 |
|  | 8 | 6.36 | 10.34 | 756.37 | 1.21 | 369.47 | 53.56 | 52.36 | 28.46 | 4.69 | 359.38 | 595.29 | 123.60 |
| TAA-Cilo100 | 1 | 4.30 | 6.30 | 567.45 | 0.63 | 164.62 | 36.84 | 48.03 | 6.56 | 6.15 | 717.24 | 570.78 | 159.93 |
|  | 2 | 3.90 | 7.25 | 532.45 | 0.76 | 360.38 | 76.00 | 28.35 | 20.43 | 5.26 | 254.79 | 589.69 | 160.60 |
|  | 3 | 4.70 | 13.15 | 523.65 | 0.92 | 271.15 | 40.28 | 31.35 | 16.36 | 6.33 | 373.60 | 680.51 | 149.79 |
|  | 4 | 3.68 | 7.85 | 563.49 | 1.06 | 364.25 | 36.02 | 43.80 | 8.68 | 6.30 | 323.73 | 727.70 | 98.80 |
|  | 5 | 4.34 | 6.57 | 530.85 | 0.66 | 290.43 | 77.37 | 78.68 | 12.35 | 5.68 | 835.17 | 648.69 | 137.82 |
|  | 6 | 3.83 | 7.18 | 455.78 | 0.77 | 347.45 | 49.47 | 46.12 | 9.88 | 6.17 | 424.45 | 576.72 | 128.06 |
|  | 7 | 4.47 | 6.64 | 582.90 | 1.00 | 207.56 | 68.46 | 39.27 | 6.76 | 7.00 | 682.38 | 668.60 | 138.65 |
|  | 8 | 4.23 | 7.39 | 572.86 | 0.79 | 265.66 | 45.49 | 37.00 | 6.69 | 5.32 | 756.63 | 576.50 | 111.80 |
